# Supplementary material for: Development of a Scalable Process of Film-Coated bi-Layer Tablet Containing Sustained-Release Metoprolol Succinate and Immediate-Release Amlodipine Besylate
Source: Pharmaceutics. 2021 Oct 27;13(11):1797. doi: 10.3390/pharmaceutics13111797 (PMC8618854; doi:10.3390/pharmaceutics13111797)
Supplement: Supplementary file 1 [file pharmaceutics-13-01797-s001.zip › pharmaceutics-1378146-supplementary.pdf]

pharmaceutics

MDPI

Supplementary Materials

Development of a Scalable Process of Film Coated bi-Layer Tablet Containing Sustained-Release Metoprolol Succinate and Immediate-Release Amlodipine Besylate

Nguyen Thi Linh Tuyen [1,*], Le Quan Nghiem [2], Nguyen Duc Tuan [2] and Phuoc Huu Le [3]

1 Faculty of Pharmacy, Can Tho University of Medicine and Pharmacy, 179 Nguyen Van Cu, Can Tho City 94000, Vietnam
2 Faculty of Pharmacy, University of Medicine and Pharmacy at Ho Chi Minh City, 41 Dinh Tien Hoang Street, District 1, Ho Chi Minh City 760000, Vietnam; lequannghiem55@gmail.com (L.Q.N.); ductuan@ump.edu.vn (N.D.T.)
3 Department of Physics and Biophysics, Faculty of Basic Sciences, Can Tho University of Medicine and Pharmacy, 179 Nguyen Van Cu, Can Tho City 94000, Vietnam; lhuuphuoc@ctump.edu.vn
* Correspondence: ntltuyen@ctump.edu.vn; Tel.: +84-91-807-1943

1. System Suitability

Table S1. Mean values of chromatographic parameters and their relative standard deviation values calculated from 6 consecutive injections. t_R is retention time, S is peak area, A_S is asymmetric factor, R_S is the resolution, N is theoretical plate numbers.

Active ingredient			t_R (min.)	S (µAU x sec.)	A_S	R_S	N
Standard mixture	Metoprolol succinate	Mean	3.533	3300978	0.8	-	16461
		RSD	0.12%	0.49%	1.27%	-	1.79%
	Amlodipine besylate	Mean	5.758	543754	1.2	6.0	24232
		RSD	0.59%	0.67%	1.49%	1.63%	1.89%
Sample	Metoprolol succinate	Mean	3.597	3017171	0.9	-	16412
		RSD	0.13%	0.10%	1.08%	-	1.81%
	Amlodipine besylate	Mean	5.845	714285	1.2	5.7	23805
		RSD	0.20%	0.21%	0.87%	0.76%	1.76%

2. The Validation of the HPLC Method

Table S2. Validation parameters of the analytical HPLC procedure.

Validation characteristics	Metoprolol succinate	Amlodipine besylate
Linearity range (µg/ml)	40–140	4–14
Regression coefficient (R^2)	$R^2 = 0.9987$	$R^2 = 0.9984$
Repeatability ($n = 6$)	RSD = 1.23%	RSD = 1.21%
Inter-day precision (n=18)	RSD = 1.45%	RSD = 1.27%
Accuracy ($n = 9$, $p < 0.05$)		
Recovery (%)	98.52%–101.14%	98.16%–99.82%
RSD	1.14%	0.63%

Validation results showed that the HPLC method was suitable, selective, high precision (RSD < 2%), and accuracy. Indeed, the recoveries were in the range of 98.0–102.0% of the expected values. Thus, the present procedure was applied for simultaneous determination of metoprolol succinate and amlodipine besylate.

3. Optimize Formulation of Film Coated Bi-Layer Tablet Containing Sustained-Release Metoprolol Succinate and Immediate-Release Amlodipine Besylate

Table S3. Variables in the experimental design for optimizing the drug formulation.

Independent Variables	Level 1	Level 2	Level 3	Level 4
X1: polymer mixture (%)	35	40	45	-
X2: ratio of diluent	S:D (2:1)	S:D (1:1)	S:A (2:1)	S:A (1:1)
X3: hardness (kp)	8–10	10–12	-	-
**Dependent variables**	Maximum			
Y1: cumulative percentage of metoprolol succinate release at 1 hour	0%–25%			
Y2: cumulative percentage of metoprolol succinate release at 4 hour	20%–40%			
Y3: cumulative percentage of metoprolol succinate release at 8 hour	40%–60%			
Y4: cumulative percentage of metoprolol succinate release at 20 hour	≥ 80%			

(with S – Starch 1500, D – Ditab, A – Avicel).

18 formulation were designed according to D-optimal model using Design Expert software (version 6.0.6, Stat-Ease Inc., Minneapolis, USA). These formulations were summarized in Table 4.

Table S4. The independent variables of 18 formulations (M1-M18).

Formula	X1	X2	X3		X1	X2	X3
M1	35%	S:D (2:1)	10–12 kp	M10	40%	S:A (1:1)	10–12 kp
M2	35%	S:D (2:1)	8–10 kp	M11	40%	S:A (1:1)	8–10 kp
M3	35%	S:A (1:1)	10–12 kp	M12	40%	S:D (1:1)	10–12 kp
M4	35%	S:D (1:1)	10–12 kp	M13	45%	S:A (2:1)	10–12 kp
M5	35%	S:A (2:1)	8–10 kp	M14	45%	S:D (2:1)	10–12 kp
M6	35%	S:A (1:1)	8–10 kp	M15	45%	S:A (2:1)	8–10 kp
M7	40%	S:A (2:1)	10–12 kp	M16	45%	S:A (1:1)	10–12 kp
M8	40%	S:A (2:1)	8–10 kp	M17	45%	S:D (1:1)	8–10 kp
M9	40%	S:D (2:1)	8–10 kp	M18	45%	S:D (1:1)	10–12 kp

Where X_1: % polymer mixture, X_2: ratio of diluent (S - Starch 1500, D - Ditab, A - Avicel), X_3: hardness.

Table S5. The metoprolol succinate release percentage of 18 formulations (M1–M18).

Formula	Cumulative Metoprolol Succinate Release Percentage (%)				Formula	Cumulative Metoprolol Succinate Release Percentage (%)			
	1 h	4 h	8 h	20 h		1 h	4 h	8 h	20 h
**M1**	18.96	44.36	64.04	85.08	M10	22.10	50.20	70.70	93.09
M2	17.03	45.23	64.71	87.77	M11	17.51	42.59	61.6	93.18
M3	20.33	48.17	73.19	94.96	M12	19.22	48.88	71.22	92.31
M4	17.21	42.24	63.66	86.18	M13	14.69	37.08	54.2	69.87
M5	17.96	43.86	65.70	85.56	M14	13.65	35.61	52.27	83.93
M6	16.33	37.81	56.44	80.35	M15	16.38	44.27	63.04	75.02
M7	15.84	39.54	57.96	89.26	M16	18.13	45.03	63.72	73.95
M8	21.29	51.34	73.68	93.27	M17	21.22	47.05	63.37	91.97
M9	15.89	39.66	59.67	85.52	M18	20.76	43.69	65.86	89.78

The data in **Table S5** were used as the inputs for BCPharSoft OPT to optimize the formulation. The results of the accuracy of model statistics from BCPharSoft OPT outputs were presented in **Table S6**. The results show that the prediction ability of the models has good reliability. Therefore, these models could be used for multivariate optimization.

Table S6. Model statistical outputs obtained from BCPharSoft.

R^2	1 h	4 h	8 h	20 h
Training	0.90	0.97	0.86	0.98
Test	0.96	0.91	0.85	0.96

Table S7. Optimal formula predicted by BCPharSoft OPT software.

	X1	X2	X3
Predicted parameters	45%	S:D (2:1)	10-12 kp

Table S8. The metoprolol succinate release percentage of the optimal formulation versus the predicted formulation ($n = 3$).

	Cumulative Metoprolol Succinate Release Percentage (%)			
	1 h	4 h	8 h	20 h
Predicted	13.75	35.53	52.05	84.03
Observed	13.90 ± 0.73	35.22 ± 1.94	52.36 ± 0.85	85.26 ± 1.40
P-values	0.751	0.808	0.591	0.268

Table S9. Dissolution test of film coated bi-layer tablet of three batches.

Active Ingredient	Batch	Cumulative Percentage Drug Release (%)				
		30 min	1 h	4 h	8 h	20 h
Metoprolol Succinate	1	-	12.78	35.28	50.43	87.39
	2	-	14.33	36.32	53.86	88.31
	3	-	14.36	34.31	52.07	85.76
	TB	-	13.82	35.30	52.12	87.15
	RSD%		0.91	1.01	1.72	1.29
Amlodipine besylate	1	96.74	-	-	-	-
	2	97.79	-	-	-	-
	3	103.43	-	-	-	-
	TB	99.32	-	-	-	-
	RSD%	2.97				
